# Twenty years of ungulate disease surveillance by the Canadian Wildlife Health Cooperative (2003–2022)

**DOI:** 10.1371/journal.pone.0343520

**Published:** 2026-03-05

**Authors:** Kerry Schutten, Leonard Shirose, Brian Stevens, Dayna Goldsmith, Owen Slater, Jamie L. Rothenburger, Susan Kutz, Stéphane Lair, Megan Jones, Laura Bourque, Scott McBurney, Margo Pybus, Iga Stasiak, Erin Moffatt, Naima Jutha, Helen Schwantje, Larissa Nituch, Damien O. Joly, Claire M. Jardine, Trent K. Bollinger

**Affiliations:** 1 Canadian Wildlife Health Cooperative, multiple locations: Abbotsford, British Columbia, Canada; Calgary, Alberta, Canada; Saskatoon, Saskatchewan, Canada; Guelph, Ontario, Canada; Saint-Hyacinthe, Québec, Canada; Prince Edward Island, Charlottetown, Canada; 2 Ontario Veterinary College, University of Guelph, Guelph, Ontario, Canada; 3 Faculty of Veterinary Medicine, University of Calgary, Calgary, Alberta, Canada; 4 Faculté de Médecine Vétérinaire, Université de Montréal, Saint-Hyacinthe, Québec, Canada; 5 Atlantic Veterinary College, University of Prince Edward Island, Charlottetown, Prince Edward Island, Canada; 6 Government of Alberta and University of Alberta, Edmonton, Alberta, Canada; 7 Government of Saskatchewan, Saskatoon, Saskatchewan, Canada; 8 Department of Environment and Climate Change, Government of the Northwest Territories, Yellowknife, Northwest Territories, Canada; 9 British Columbia Ministry of Water, Lands and Resource Stewardship (Emeritus), Victoria, British Columbia, Canada; 10 Ontario Ministry of Natural Resources, Peterborough, Ontario, Canada; 11 Western College of Veterinary Medicine, University of Saskatchewan, Saskatoon, Saskatchewan, Canada; Cairo University Faculty of Veterinary Medicine, EGYPT

## Abstract

Free-ranging wild ungulates are integral to the health and well-being of Canadian socioecological systems, contributing various One Health benefits (e.g., nutrient cycling, sustainable food resources) to the people and other animals that coexist with them. In North America, ungulates face a range of threats to their population health. To address knowledge gaps surrounding the health of Canadian ungulates, we conducted a retrospective analysis of 20 years of ungulate morbidity and mortality data collected through passive disease surveillance conducted by the Canadian Wildlife Health Cooperative (CWHC) and through submissions from collaborative partners. In total, 2525 cases across 12 species were assigned a category of diagnosis (COD) by a CWHC veterinary pathologist. Infectious/ inflammatory/ transmissible CODs accounted for 53.0% of all cases, with two diagnoses made most frequently: chronic wasting disease (CWD) and *Parelaphostrongylus tenuis* (*P. tenuis*) infection. We identified a significant increase in the proportion of cervid cases diagnosed with CWD in Saskatchewan that was consistent across species, with the odds of an individual cervid in Saskatchewan being CWD positive increasing by 22% per year. We also detected a significant increase in the proportion of moose (*Alces americanus*) cases diagnosed with *P. tenuis*, and this trend was consistent across endemic regions in Canada. Emerging diseases were also detected for the first time through our surveillance approach (e.g., the first Ontario cases of epizootic hemorrhagic disease virus, detected in white-tailed deer (*Odocoileus virginianus*)). Trauma (15.3%) and emaciation (8.9%) were the most frequently assigned non-infectious CODs. We highlight potential disease threats to SAR that may emerge secondary to changing distributions of sympatric ungulate species and the pathogens they carry (e.g., CWD positive deer/elk within known caribou ranges in Saskatchewan). Our results highlight the strengths of passive disease surveillance, as well as the need for an integrated, holistic wildlife surveillance approach in Canada.

## Introduction

Wild ungulates are integral to the health of animals, people, and the environment, contributing various One Health benefits across their endemic distributions. Ungulate species contribute to the balance of many important ecosystem functions in Canada and throughout North America more broadly, through their browsing behaviour and movement ecology (e.g., nitrogen cycling in forest ecosystems and modification of plant community structures) and as prey species for large mammalian predators [1–4]. Ungulate species also play an important role in the food security and cultural practices of many Indigenous communities living within Canada [5–7]. The opportunity to view native ungulates in the wild or to participate in recreational hunting are both important incentives for many Canadians as well as tourists visiting Canada, providing significant benefit to the Canadian economy [8,9]. There are eleven ungulate species native to Canada across three families: Cervidae (moose (*Alces americanus*), elk (*aka* Wapiti; *Cervus canadensis*), mule deer (*Odocoileus hemionus*), white-tailed deer (*Odocoileus virginianus*), and caribou (*Rangifer tarandus*); Bovidae (bison (*Bison bison*), mountain goat (*Oreamnos americanus*), muskox (*Ovibos moschatus*), bighorn sheep (*Ovis canadensis*) and Dall’s sheep (*Ovis dalli*)); and the sole extant member of the Antilocapridae family, the pronghorn (*Antilocapra americana*).

Ungulate species in Canada face numerous, potentially synergistic threats to population health, and understanding cumulative drivers of morbidity and mortality can be challenging given the many logistical and financial constraints associated with sample collection and necropsy investigation of large mammals distributed across very wide and often remote geographic ranges [10]. Despite this paucity of data, several species have been demonstrated to be in decline both regionally and across their North American distributions. In Canada, bison occupy a fraction of their initial range [11], with wood bison (*Bison bison athabascae*) listed federally as a species at risk (SAR) [12]. Similarly, many Canadian caribou populations (several ecotypes and subspecies) are in decline across Canada and are listed as SAR variably across federal, provincial, and territorial legislations [12]. Moose in Canada are also experiencing regional declines (e.g., populations within the Boreal Plain ecozone in Saskatchewan and Manitoba), with Eastern moose (*Alces americanus americanus*) provincially protected as a SAR on mainland Nova Scotia [13–16]. Conversely, white-tailed deer populations in some parts of Canada have experienced northern and western range expansion in the past century [17,18], though they are still susceptible to infectious and non-infectious health stressors.

Anthropogenic stressors such as climate change, habitat fragmentation and loss, increasing urbanization, ungulate-vehicle collisions, and overharvesting can all have negative implications for the individual- and population-level health of ungulate species within Canada and across North America [10,19–21]. Infectious diseases can also become a driver of population decline, and may serve as a barrier to population recovery for some species. The negative impacts of disease on ungulate population health may be further exacerbated by the effects of climate change and landscape change, resulting in shifting ranges of hosts (such as white-tailed deer) and their pathogens, or the introduction of novel diseases to previously naïve sympatric ungulate populations [22,23]. Some diseases of concern affecting ungulate SAR and sympatric species in Canada include chronic wasting disease (CWD), bovine tuberculosis, brucellosis, *Parelaphostrongylus tenuis* infection (*P. tenuis*, *aka* meningeal worm), and emaciation secondary to high winter tick (*Dermacentor albipictus*) burden [10,23–27]. Infectious respiratory diseases, such as bronchopneumonia caused by *Mycoplasma ovipneumoniae* infection, have also had serious and potentially population-limiting impacts on free-ranging wild sheep and goat species in Western North America [28–30]. Infectious diseases can lead to individual-level morbidity and mortality (e.g., ataxia, altered mentation, emaciation, and recumbency in CWD-positive cervids [31]), but may also have population-level impacts (e.g., reduced survival and fecundity in wood bison positive for bovine tuberculosis and brucellosis [25], or increased moose calf mortality during winter tick epizootic events [26]). Infectious and non-infectious stressors often occur synergistically, increasing pressure on populations (e.g., low trace-element status identified as an additional contributor to reduced survival in caribou alongside *Brucella suis biovar 4* *and* *Erysipelothrix rhusiopathiae* [22])*.*

Wildlife disease surveillance may involve either passive (*aka* scanning) or active (*aka* targeted) surveillance approaches, or a combination of both [32]. Passive wildlife disease surveillance allows researchers to maintain a continuous watch over the health of endemic wildlife, and provides opportunity for the early identification of emerging and re-emerging concerns [33]. Conversely, active surveillance approaches target specific individuals or diseases in response to an issue of concern [33]. The Canadian Wildlife Health Cooperative (CWHC) is a collaborative organization made up of wildlife health experts, and one of its mandates is to provide long-term passive wildlife disease surveillance in Canada [34]. The CWHC includes six regional centres (based at the five veterinary colleges in Canada located in Alberta, Saskatchewan, Ontario, Québec and Prince Edward Island, plus the government of British Columbia’s Inter-ministry Wildlife Health Group) that offer veterinary diagnostic services for wildlife. The CWHC is also supported through collaborative partnerships with many organizations across Canada.

We aimed to address knowledge gaps surrounding the health of wild ungulate species in Canada by describing the temporal and spatial trends in morbidity and mortality detected as part of ongoing passive wildlife disease surveillance conducted by the CWHC and through submissions from collaborative partners. Our objectives were to 1) conduct a retrospective analysis of 20 years of ungulate morbidity and mortality data (2003–2022) collected by the CWHC during passive wildlife disease surveillance, and 2) assess patterns of risk associated with infectious and non-infectious diseases in Canadian ungulates, with a particular focus on SAR (caribou, bison, and moose). We expected to detect increasing trends in CWD in our dataset consistent with recent active surveillance programs in Canada [27,35], though we predicted our samples would be biased to Saskatchewan due to regional differences in wildlife disease surveillance reporting. We also expected to detect an increasing temporal trend in *P. tenuis* cases in endemic regions, particularly in moose where the parasite is a known important driver of population decline [13,23]. Finally, we discuss the strengths and limitations of passive wildlife disease surveillance and provide recommendations for an integrated ungulate disease surveillance approach in Canada.

## Materials and methods

### Case submission and data extraction

Carcasses or biological samples from free-ranging ungulates were submitted for post-mortem examination by veterinary pathologists at one of the six CWHC regional diagnostic centres between1 January 2003 and 31 December 2022. Carcasses and samples typically originated from partnering agencies including government and community-based wildlife health surveillance programs, but members of the public, hunters, and rehabilitation facilities were also sources of submissions. Cases submitted as part of the passive disease surveillance program included all eleven native ungulate species in Canada: moose, elk, mule deer, white-tailed deer, caribou, bison, mountain goat, muskox, bighorn sheep, Dall’s sheep, and pronghorn, as well as introduced but free-ranging fallow deer (*Dama dama*).

Records for all ungulate cases were extracted from the CWHC database on 9 November 2023. To be included in our retrospective analysis, cases needed complete date, location, and species information, as well as sufficient sample quality and associated case history to allow the pathologist to determine a category of diagnosis. Domestic ungulates (e.g., horses, cows, small ruminants), zoo animals, and farmed wildlife were also excluded from the analysis. Escaped farmed and/or non-native wild ungulate species (i.e., fallow deer) were retained in the analysis provided there was sufficient case history to determine that they were truly free-ranging (e.g., feral). Cases that were part of active surveillance programs (e.g., active surveillance for CWD through hunter submissions of apparently healthy animals) were excluded from the dataset.

### Category of diagnosis

The details of each case were evaluated to assign an overall category of diagnosis (COD). In total, 2525 cases had sufficient data to be assigned a COD. Cases included those that had a full carcass for necropsy as well as those with selected tissues submitted for analysis, provided the pathologist determined the samples submitted were sufficient to determine a diagnosis based on the presenting complaint and history. Mortality states for cases ranged from hunter-harvested, euthanasia following observed morbidity, or found dead. The provided history, clinical signs, gross postmortem examination, histopathology, and applicable ancillary tests were integrated as part of the pathologists’ diagnostic evaluation and interpretation of each case.

Six primary CODs were considered: infectious/inflammatory/transmissible diseases, emaciation, trauma, other, normal, and open/no diagnosis (see Table 1 for definitions). The primary COD represents the principal cause of morbidity, mortality, or lesions identified in each case, based on the pathologist’s interpretation as described above. Subcategories were also included for some CODs (Table 1). For simplicity, the six CODs were grouped during the data analysis phase: infectious/inflammatory/transmissible cases were considered separately and are hereafter referred to as “infectious CODs”, while trauma, emaciation, and other cases were grouped together as “non-infectious CODs” (Table 1). Importantly, trauma was never defined as the primary COD for euthanasia (e.g., captive bolt, gunshot) or hunter-harvested submissions. Normal and open/no-diagnosis cases were excluded from most of the analysis but are included in the descriptive statistics as they represent the broader geographic and temporal scale of the CWHC passive disease surveillance program. Within infectious CODs, cases were assigned a body system (see S1 Methods) and a primary pathogen when identified. Additional and/or incidental pathogens were sometimes detected across all cases (i.e., pathogens were described as incidental based on the pathologist interpretation of species/lesion/pathogen and did not impact the COD classification).

**Table 1:** Category and subcategories of diagnosis for ungulate cases.

	Category of Diagnosis	Definition	Subcategory of Diagnosis (N/A = Not Applicable)
***Infectious***	**Infectious/ Inflammatory/ Transmissible**	The primary diagnosis was of infectious, inflammatory or transmissible in origin, based on history, necropsy findings and additional testing	Bacterial, Degenerative, Fungal, Other, Parasitic, Prion, Unknown, Viral
***Non-infectious diagnostic categories***	**Emaciation**	The primary diagnosis was emaciation based on history, necropsy findings and additional testing	Winter tick, otherwise N/A
	**Other**	Less common categories of diagnosis (see subcategory), based on history, necropsy findings and additional testing	Fetal Distress, Neoplasia, Nutritional, Other, Toxicity
	**Trauma**	The primary diagnosis was trauma based on history, necropsy findings and additional testing. Trauma was never defined as the primary COD for euthanasia (e.g., captive bolt, gunshot) or hunter-harvested submissions.	Capture-Related, Drowning, Other, Predation, Unknown, Vehicular
***Normal or Non-Diagnostic***	**Normal**	Carcass and/or samples plus history indicated animal was healthy at the time of death (e.g., normal, hunter-killed animal; see S1 Methods for additional categorization details)	N/A
	**Open/ No Diagnosis**	Cases which had a full necropsy or adequate sampling and testing as per the pathologist, but necropsy and/or testing were inconclusive	N/A

Categories of diagnosis (COD) and subcategories for ungulate cases submitted for passive disease surveillance to the Canadian Wildlife Health Cooperative between 2003 and 2022. CODs are also grouped more broadly as infectious, non-infectious, or as normal/non-diagnostic.

Diagnosing winter tick as the cause of emaciation involves a combination of associated clinical findings (e.g., high tick numbers, visible hair loss, anemia) in addition to ruling out other causes of emaciation. Therefore, these cases received a primary COD of “emaciation” with a subcategory of “winter tick” when winter ticks were presumed or suspected as a significant factor in emaciation. For cases where winter tick was identified on the submitted carcass or sample but the significance of the tick burden could not be determined, winter tick was listed as an additional pathogen and not considered as part of the primary COD. *Parelaphostrongylus tenuis* was similarly assigned as the primary cause of infectious COD when the pathologist had sufficient evidence of lesions in the brain consistent with *P. tenuis* damage, along with typical clinical signs or history, including cases where the parasite itself was not identified on histopathology. *Parelaphostrongylus tenuis* was considered an additional or incidental finding in cases when there was no evidence of disease from parasite migration (this typically occurred in the definitive host species, white-tailed deer).

### Demographic data

Given the opportunistic nature of passive surveillance data, necropsy submissions were not always assigned a specific subspecies. As such, our analysis was conducted at the species level only. Cases were further categorized by sex (male, female, unknown) and age (adult, juvenile (i.e., not reproductively active), unknown), as determined by the pathologist.

### Statistical analysis and mapping

All statistical analyses were performed using R (version 4.4.1; R Core Team, 2022). For all analyses, a significance level of α = 0.05 was assigned, and Clopper-Pearson exact 95% confidence intervals were calculated for proportions.

We fit separate binomial mixed-effects logistic regression models to assess changes in the proportion of both CWD and *P. tenuis* positive cases over time, including year as the fixed effect (package “lme4”, “glmer” function; [36]). To account for interspecific variation in disease status in the CWD analysis, we included species as a random effect. Due to differences in wildlife surveillance reporting within other CWD-endemic provinces, CWD-positive cases within our dataset were restricted to Saskatchewan. We therefore focused the CWD analysis to include only cases originating from Saskatchewan, and including only CWD-susceptible species (i.e., cervids: elk (n = 98), moose (n = 303), mule deer (n = 453), and white-tailed deer(n = 456)). Fallow deer and caribou from Saskatchewan were excluded from this analysis due to low sample size (n = 1 for both species). For our *P. tenuis* model, we included region as a random effect and restricted our analysis to moose cases from *P. tenuis-*endemic regions of Canada.

Model fit evaluations were performed to assess overdispersion, collinearity, and residual patterns using the packages *performance and DHARMa* [37,38]*.* A likelihood ratio test was applied for both analyses to compare the simpler random-intercept model with the random-intercept-slope model [36]. In both cases, adding random slopes to allow for differences in species-temporal (CWD) or regional-temporal (*P. tenuis*) trends did not improve overall model fit.

Maps were generated using QGIS (version 3.38.3-Grenoble; QQIS Development Team, 2024). The SAR distribution range maps are available online from the Environment and Climate Change Canada Data catalogue; the priorityspecies.gdb file was used in our maps with data updated in November 2023 [39].

## Results

**Fig 1:**
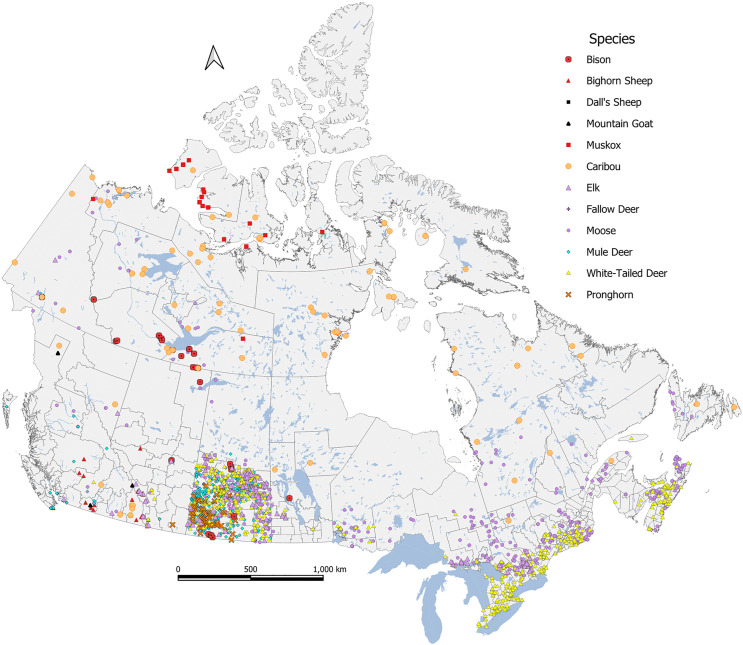
Distribution of ungulate cases across Canada. The distribution of ungulate cases (N = 2525) submitted to the Canadian Wildlife Health Cooperative’s passive wildlife disease surveillance program between 2003−2022. Scientific names for all species are described in text and in Table 2. Basemap Adapted from Statistics Canada, Boundary Files Catalogue no. 92-160-G ISBN 978-1-100-19466-0, November 2011. This does not constitute an endorsement by Statistics Canada of this product.

A total of 2525 cases across 12 species met our inclusion criteria and were assigned a COD (Table 2). Infectious CODs were reported for 53.0% of all ungulate cases, with non-infectious CODs making up a further 32.4% of cases. Normal and open/no diagnosis cases were assigned as the COD in 10.6% and 4.0% of cases, respectively. White-tailed deer were the most frequently represented species and made up 30.6% of cases (n = 772), followed by moose (n = 652; 25.8%; Table 2). Across all species, males constituted 46.7% of cases (n = 1179) followed by females (n = 990; 39.2%) and individuals of unknown sex (n = 356; 14.1%; S1 Table). The majority of ungulate cases were adults (n = 1605; 63.6%), followed by juveniles (n = 495; 19.6%) and individuals of unknown age (n = 425; 16.8%; S2 Table). Cases were distributed across Canada, with regional differences in sample submission reflected by increased density of samples from Saskatchewan and Ontario (Fig 1).

**Table 2:** Summary statistics for all ungulate cases organized by primary category of diagnosis.

Primary Category of Diagnosis	*Infectious/ Inflammatory/ Transmissible*	*Emaciation*	*Other*	*Trauma*	*Normal*	Open/ No Diagnosis	*Total for Species*
**Species** (*Scientific Name*)	**Cases (%)**	**Cases (%)**	**Cases (%)**	**Cases (%)**	**Cases (%)**	**Cases (%)**	**Cases (%)**
**White-tailed Deer** (*Odocoileus virginianus*)	301 (39.0)	56 (7.3)	108 (14.0)	161 (20.9)	97 (12.6)	49 (6.4)	**772 (30.6)**
**Moose** (*Alces americanus*)	407 (62.4)	73 (11.2)	37 (5.7)	49 (7.5)	65 (10.0)	21 (3.2)	**652 (25.8)**
**Mule Deer** (*Odocoileus hemionus*)	329 (65.5)	37 (7.4)	14 (2.8)	78 (15.5)	33 (6.6)	11 (2.2)	**502 (19.9)**
**Elk** (*Cervus canadensis*)	110 (49.3)	17 (7.6)	6 (2.7)	46 (20.6)	40 (17.9)	4 (1.8)	**223 (8.8)**
**Caribou** (*Rangifer tarandus*)	88 (50.9)	29 (16.8)	28 (16.2)	12 (6.9)	13 (7.5)	3 (1.7)	**173 (6.9)**
**Bighorn Sheep** (*Ovis canadensis*)	17 (29.8)	2 (3.5)	3 (5.3)	31 (54.4)	3 (5.3)	1 (1.8)	**57 (2.3)**
**Pronghorn** (*Antilocapra americana*)	27 (50.9)	3 (5.7)	9 (17.0)	2 (3.8)	8 (15.1)	4 (7.6)	**53 (2.1)**
**Bison** (*Bison bison*)	30 (62.5)	5 (10.4)	2 (4.2)	4 (8.3)	5 (10.4)	2 (4.2)	**48 (1.9)**
**Muskox** (*Ovibos moschatus*)	21 (67.7)	2 (6.5)	0 (NA)	0 (NA)	2 (6.5)	6 (19.4)	**31 (1.2)**
**Dall’s sheep** (*Ovis dalli*)	6 (75.0)	0 (NA)	0 (NA)	1 (12.5)	0 (NA)	1 (12.5)	**8 (0.3)**
**Mountain Goat** (*Oreamnos americanus*)	2 (50.0)	0 (NA)	0 (NA)	2 (50.0)	0 (NA)	0 (NA)	**4 (0.2)**
**Fallow Deer** (*Dama dama*)	0 (NA)	0 (NA)	0 (NA)	1 (50.0)	1 (50.0)	0 (NA)	**2 (0.1)**
**Total**	**1338 (53.0)**	**224 (8.9)**	**207 (8.2)**	**387 (15.3)**	**267 (10.6)**	**102 (4.0)**	**N/A (N/A)**

All ungulate cases (n = 2525) submitted for passive disease surveillance to the Canadian Wildlife Health Cooperative between 2003 and 2022, organized by species and primary category of diagnosis (COD).

### Infectious categories of diagnosis

**Fig 2:**
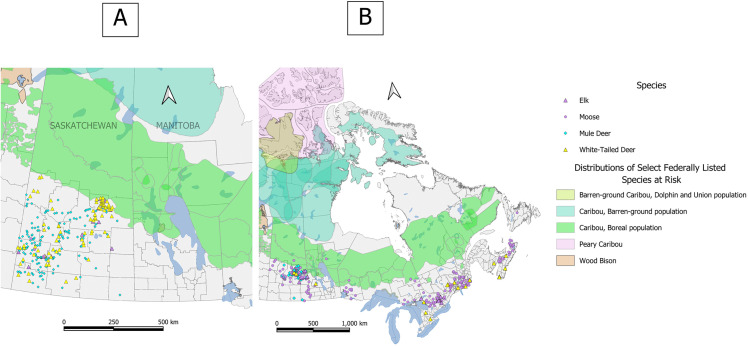
Spatial distribution of CWD and *P. tenuis* cases. Distribution of cases submitted for passive disease surveillance to the Canadian Wildlife Health Cooperative between 2003 and 2022 and diagnosed with A) chronic wasting disease (CWD; N = 297) and **B)** *P. tenuis* (N = 277). Distributions of species federally regulated as species at risk in Canada are also demonstrated. Scientific names for all species are described in text and in Table 2. Basemap adapted from Statistics Canada, Boundary Files Catalogue no. 92-160-G ISBN 978-1-100-19466-0, November 2011; and Priority Species for Species at Risk, ECCC Data Catalogue c3d1771f-fa10-4a09-ba6e-dece0ceb5f37, December 2023. This does not constitute an endorsement by Statistics Canada of this product.

A total of 1338 of 2525 (53.0%) cases were categorized as infectious in origin (Table 2). Within infectious CODs, 57 different pathogens were diagnosed as the causative agent for the primary infectious COD (S3 Table). Overall, parasites were the most frequently diagnosed pathogen within infectious CODs (N = 469; 35.1% of infectious cases), followed by bacterial pathogens (n = 366, 27.4% of infectious cases) and prion disease (n = 297; 22.2% of infectious cases; Table 3). Two pathogens constituted the majority of primary infectious diagnoses: CWD (n = 297; 22.2% of infectious cases) and *P. tenuis* (n = 277; 20.7% of infectious cases; S3 Table). CWD-positive submissions were restricted to Saskatchewan, while cases diagnosed with *P. tenuis* were distributed across the known endemic regions for this parasite within Canada (Fig 2). Six pathogens were detected which are federally monitored under the Health of Animals Act and/or Reportable Diseases Regulations due to their strong potential to impact human and animal health and the Canadian economy [40,41]: the causative prion agent of CWD, *Besnoitia sp.,* the causative agent of anthrax (*Bacillus anthracis*), *Brucella suis* (a causative agent of brucellosis; confirmed as biovar 4 when ancillary testing was performed), Epizootic Hemorrhagic Disease Virus (EHDv), and the causative agent of bovine tuberculosis (*Mycobacterium bovis*; S4 Table). Additional and/or incidental pathogens detected are provided in S5 Table (e.g., incidental findings of *Sarcocyst* sp. in 115 cases).

**Table 3:** Ungulate cases by category and subcategory of diagnosis.

Primary Category Of Diagnosis	Number of Cases	Subcategory Proportion (%) of Primary COD
Subcategory		
**Infectious/ Inflammatory/ Transmissible**	**1338**	
Parasitic	469	35.1
Bacterial	366	27.4
Prion	297	22.2
Unknown	126	9.4
Viral	43	3.2
Degenerative	14	1.1
Fungal	12	0.9
Other	11	0.8
**Emaciation**	**224**	
Winter tick	33	14.7
**Trauma**	**387**	
Vehicular	133	34.4
Unknown	106	27.4
Predation	54	14.0
Other	46	11.9
Capture-Related	30	7.8
Drowning	18	4.7
**Other**	**207**	
Nutritional	64	30.9
Other	53	25.6
Neoplasia	34	16.4
Fetal Distress	34	16.4
Toxicity	22	10.6
**Normal**	**267**	
**Open/No Diagnosis**	**102**	
**Grand Total**	**2525**	

All ungulate cases (n = 2525) submitted for passive disease surveillance to the Canadian Wildlife Health Cooperative between 2003 and 2022, organized by primary category of diagnosis (COD), with subcategories of diagnosis where applicable.

**Fig 3:**
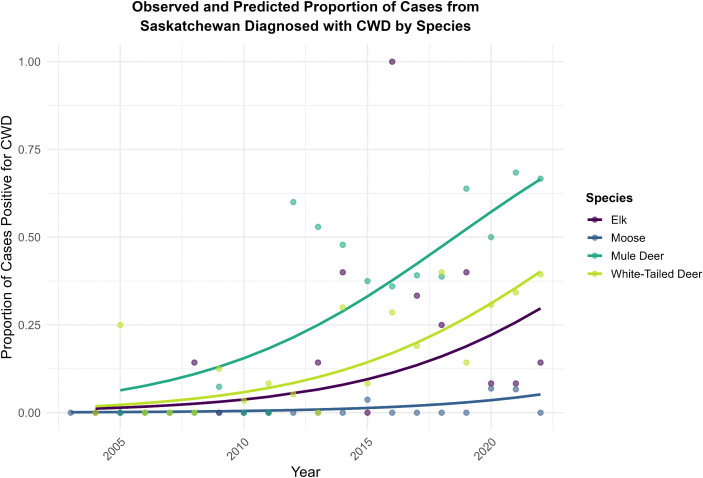
Regression analysis of CWD in cervid cases from Saskatchewan over time. Species-specific temporal trends for the observed vs predicted proportion of cervid cases (excluding fallow deer (n = 1) and caribou (n = 1)) from Saskatchewan diagnosed with chronic wasting disease (CWD) as part of the Canadian Wildlife Health Cooperative’s passive disease surveillance program between 2003 and 2022. Points indicate the observed proportions of CWD-positive cases per year. Coloured lines indicate the predicted probabilities from the mixed effects logistic regression model, by species. The model indicated a significant increasing trend (p < 0.001), which was consistent across species.

We detected a significant increase in the total proportion of CWD-positive cervid cases from Saskatchewan over time (binomial mixed-effects logistic regression analysis; β = 0.1999, p < 0.001, odds ratio (OR)=1.22), with the odds of an individual cervid being CWD positive increasing by approximately 22.0% per year. While the predicted prevalence of CWD differed significantly across the four species included in the model, adding a random slope failed to improve model fit (χ² = 1.36, *p* = 0.51, Fig 3). Therefore, we found no evidence that the proportion of CWD-positive cases was increasing differently across susceptible species in Saskatchewan.

**Fig 4:**
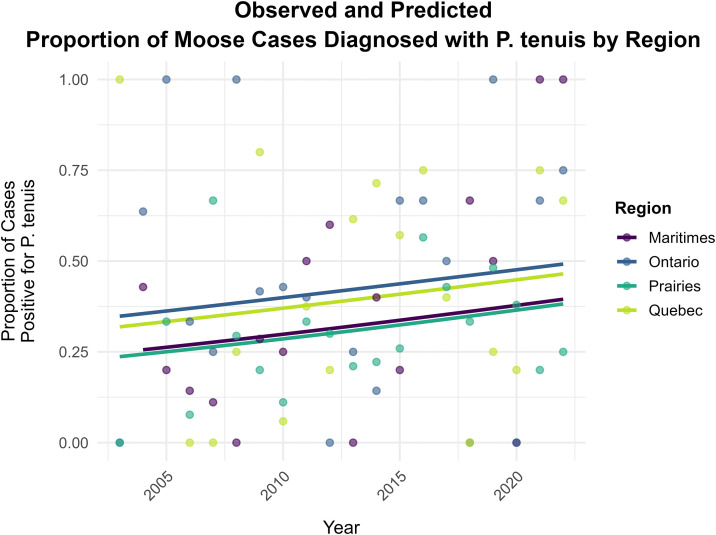
Regression analysis of *P. tenuis* positive moose cases from endemic regions. Regional-specific temporal trends for the observed vs predicted proportion of moose cases from endemic regions of Canada that were diagnosed with *Parelaphostrongylus tenuis* (*P. tenuis*) as part of the Canadian Wildlife Health Cooperative’s passive disease surveillance program between 2003 and 2022. Points indicate the observed proportions of *P. tenuis*-positive cases per year. Coloured lines indicate the predicted probabilities from the mixed-effects logistic regression model, by region. The model indicated a significant increasing trend (p = 0.047), which was consistent across all regions.

We restricted our analysis of *P. tenuis* to include only moose, given this species represented 76.5% of all primary COD *P. tenuis* cases. Cases were grouped by provincial boundary and collapsed regionally due to limited sample sizes and similar geography as follows: Ontario (n = 99), Québec (n = 110), Prairies (n = 307, including: Manitoba (n = 4) and Saskatchewan (n = 303)), and Maritimes (n = 84, including: New Brunswick (n = 15), Newfoundland and Labrador (n = 10) and Nova Scotia (n = 59)). The proportion of moose cases from endemic areas that had a diagnosis of *P. tenuis* increased significantly over time (binomial mixed-effects logistic regression analysis; β = 0.034, p = 0.047, OR=1.035) indicating that the odds of an individual moose from a *P. tenuis*-endemic region having a positive diagnosis increased by approximately 3.5% per year. There was no evidence of a regional difference in the positive *P. tenuis* temporal trend (χ² = 0.02, *p* = 0.99, Fig 4).

### Non-infectious categories of diagnosis

Of non-infectious CODs, trauma was the most frequently reported cause of morbidity and mortality (15.3% of all cases), followed by emaciation (8.9%) and other (8.2%; Table 2). Within the COD of trauma, each case was assigned one of six potential subcategorizations, and vehicular trauma cases were the most frequently represented within this category (n = 133, 34.4% of trauma cases; Table 3). A total of 33 cases were assigned a primary COD of emaciation secondary to winter tick disease (Tables 1 and S3), with a further 35 cases where winter tick was diagnosed as a pathogen of incidental or unknown significance (S4 Table).

The final non-infectious category of “Other” included five potential subcategories (Table 3). Nutritional causes were the most frequently assigned subcategory, constituting 30.9% of cases assigned an “Other” COD (n = 64; Table 3). Primary nutritional causes of morbidity and mortality were varied (see S1 Methods for full list), but the most frequently represented causes were ruminal acidosis (i.e., grain overload; n = 42) and polioencephalomalacia (n = 16). Neoplasia was diagnosed in 34 cases across the twenty years of our retrospective analysis, and toxin exposure was diagnosed in a further 22 cases, with carbamate or organophosphate poisoning detected most frequently (n = 11; Table 3; see S1 Methods for all toxin diagnoses). A subcategory of diagnosis of fetal distress was attributed to 34 cases (Table 3). In almost all cases assigned this subcategory, the fetus was submitted alone without any additional history regarding the health of the dam, and there were no cases where the dam and fetus were submitted together for necropsy investigation.

### Species at risk

**Fig 5:**
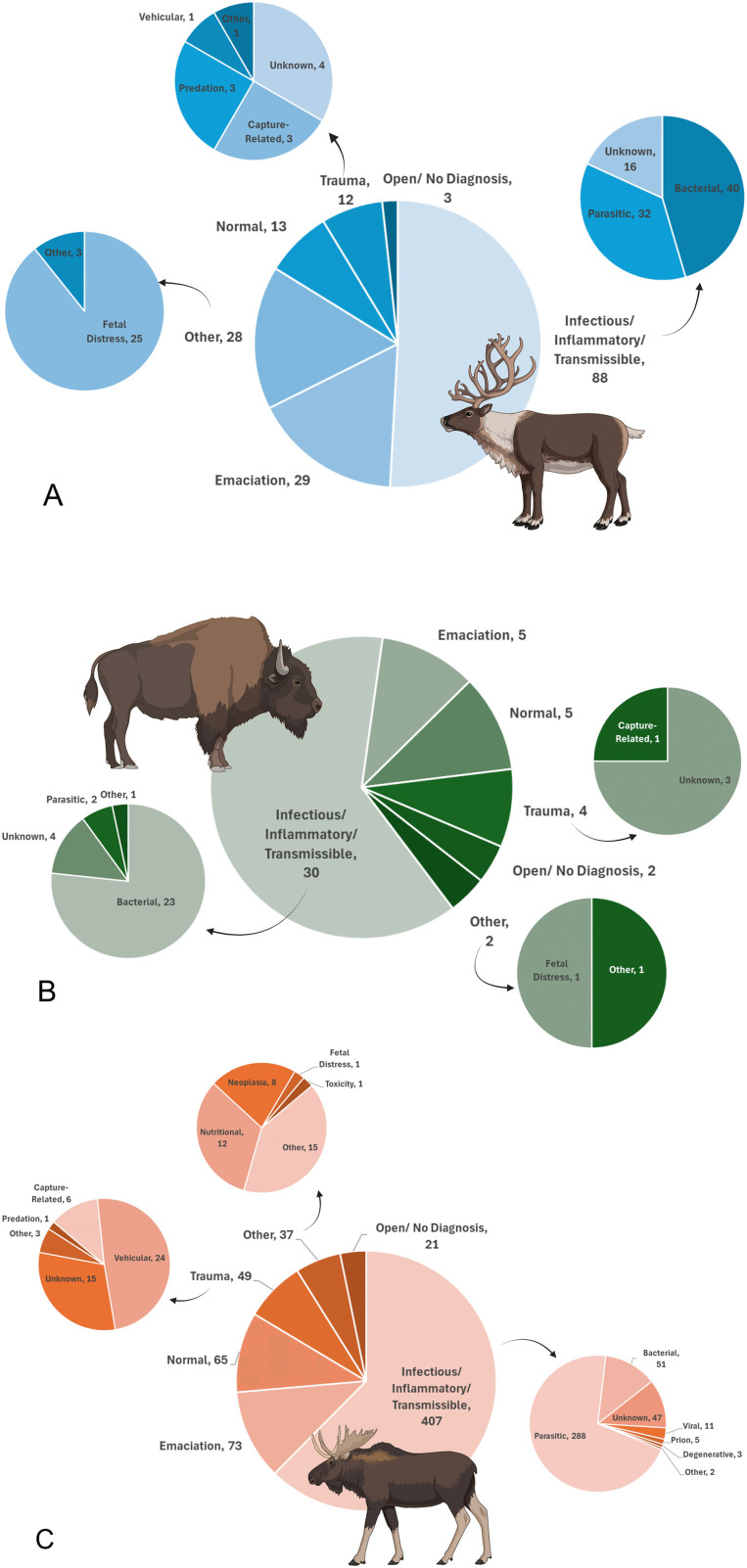
Species at risk cases by category of diagnosis. The total number of species at risk (SAR) cases submitted for passive disease surveillance to the Canadian Wildlife Health Cooperative between 2003 and 2022, by species and primary category of diagnosis: A) caribou (*Rangifer tarandus*; n = 88), B) bison (*Bison bison*; n = 30), and C) moose (*Alces americanus*; n = 652). Smaller pie charts illustrate the subcategories of diagnosis where applicable. Created in BioRender. Schutten, K. (2025). https://BioRender.com/oyb1tk3.

Caribou made up 6.9% of all cases in our dataset (n = 173), and the majority of caribou cases were assigned an infectious COD (n = 88; 50.9%; Table 2). The most frequently detected primary pathogens were bacterial in origin, followed by parasitic (Fig 5A). The most common bacterial diagnosis in caribou was *Brucella suis* (confirmed or presumed biovar 4; n = 9 confirmed, 2 suspected; S4 Table) followed by bacterial infection of unknown origin (n = 10) and mixed bacterial infections resulting in foot rot (n = 9). All 16 confirmed cases and four suspected cases of *Besnoitia tarandi* captured by our analysis were diagnosed in caribou (S4 Table), which was also the most frequently reported parasitic diagnosis in this species. Following infectious diagnoses, emaciation (n = 29) and fetal distress (subcategory of “other”, n = 25) were the next most frequently represented COD for caribou (Fig 5A). For these categories, focal years of importance were detected in the data, with 23 of 29 emaciation diagnoses occurring in 2016, and 22 of 25 fetal distress cases occurring across 2010 (n = 7) and 2011 (n = 15).

Bison represented 1.9% of cases captured by our dataset (n = 48), and the majority of bison cases were assigned an infectious COD (n = 30, 62.5%, Table 2). Bacteria were again the most frequently assigned subcategory of diagnosis (Fig 5B). A total of nine cases of anthrax were diagnosed in bison (three from 2008 and six from 2010), as was one suspected *Brucella* sp*.* case (S4 Table). An additional 53 bison and one moose were included in the CWHC database as suspected anthrax cases from the same 2010 cluster of cases in the Northwest Territories, but were not tested and are, therefore, not included in our primary analysis [42] (S4 Table).

Moose were the second-most represented species in our dataset, with 59 of 652 cases submitted from Nova Scotia where they are a provincially listed SAR ([13]; Table 2, Fig 1). Infectious diseases were again the most frequently assigned COD (n = 407, 62.4%), with *P. tenuis* cases representing the primary pathogen in the majority of infectious diagnoses (Figs 4 and 5C). Emaciation made up a significant proportion of the non-infectious CODs in moose (n = 73, 45.9% of non-infectious CODs; Fig 5C), with all 33 cases where winter tick disease was the presumed cause of emaciation diagnosed in moose. Where trauma was diagnosed as the primary cause of morbidity or mortality, vehicular trauma was the most frequently assigned subcategory (n = 24, Fig 5C).

## Discussion

### Notable causes and trends in ungulate morbidity and mortality

The results of our study reflect two decades of collaborative, cross-disciplinary and interagency ungulate passive disease surveillance. With over 2500 cases across 12 species included in our analysis, we were able to identify a range of both infectious and non-infectious causes of morbidity and mortality, many of which are not regularly reported in the literature. We also highlighted focal years of disease and mortality events in SAR and identified significant increasing temporal trends in two diseases that can have known population-limiting impacts on some ungulate species (CWD and *P. tenuis*).

CWD is an introduced, fatal prion disease of cervids which has had mounting negative impacts on cervid population health [27]. First detected among wild cervids in Canada in Saskatchewan in 2000, we have since seen extensive spread throughout Saskatchewan and Alberta, and more recent geographic spread throughout North America with detections in wild ungulates in south-western Manitoba (2021) and British Columbia (2024) [43,44]. In Saskatchewan, we identified a significant increasing temporal trend in the proportion of cervid cases diagnosed with CWD, which was similar across all four included species (Fig 3). While this trend must be interpreted with caution considering the biases of our passive surveillance program (e.g., we are testing sick/dead animals which are not representative of healthy free-ranging populations), it is consistent with increasing prevalence detected through active surveillance programs [27,35] and reflects an important shift in the threats facing cervids in Canada, including SAR. CWD has not been detected in North American caribou, yet they are presumed susceptible to infection based on genetic analyses and documented CWD cases in free-ranging European reindeer (*Rangifer tarandus tarandus*; [23]). Additionally, there is concern that habitat alteration favoured by white-tailed deer coupled with the northward range expansion of this species may increase the risk of direct and indirect contact between caribou and CWD-infected deer [17,18,24]. As illustrated in Fig 2A, we detected CWD in both deer and elk in Saskatchewan at the southern limit of the boreal caribou (*Rangifer tarandus caribou*) range, providing evidence that this risk does exist on the landscape. Passive surveillance data has revealed and continues to uncover new information about the epidemiology of CWD in Canada, in addition to improving early detection across the landscape. The first known case of CWD in Alberta was detected via passive wildlife surveillance [45], and our retrospective analysis includes the first detections of CWD in moose in Saskatchewan. These detections help inform and improve active surveillance programs in CWD-endemic areas, where we can use novel detections to target populations and geographic ranges of concern. If CWD spills over into caribou populations, passive surveillance will be a critical tool for enhancing early identification and informing active surveillance efforts.

Beyond CWD, we identified several notable infectious disease threats facing SAR and sympatric ungulate species in Canada, including a significant increase in the proportion of moose cases diagnosed with *P. tenuis* over time (Fig 4). There is ongoing concern that *P. tenuis* can lead to moose population decline, particularly in areas where high densities of the definitive *P. tenuis* host (white-tailed deer) and warmer and milder winters enhance the conditions for high parasite burden on the landscape [13,23]. *Parelaphostrongylus tenuis* may also become an important pathogen for caribou in Canada. While not detected in caribou in our surveillance data, we identified cases in deer, elk and moose at the southern edge of the boreal caribou range (Fig 2B). Winter tick populations are similarly likely to increase on the landscape, with climate change favouring both deer expansion and tick survivability in habitats suitable for moose [46,47]. High tick burdens are known to have negative impacts on moose (e.g., hair loss, anemia, disrupted feeding and mentation, emaciation [13,48,49]), and are a suspected cause of hair loss and morbidity in some caribou populations [50]. A primary COD of emaciation secondary to winter tick disease represented approximately 5% of all moose cases submitted nationally to the CWHC over our data collection period (Fig 5C).

Through the CWHC’s passive surveillance program, the first cases of epizootic hemorrhagic disease virus in Ontario ungulates were detected, enhancing our understanding of the northern expansion of orbiviruses in Canada and highlighting the usefulness of passive surveillance for early detection of emerging pathogens (S4 Table; [51]). Similarly, the first cases of adenovirus hemorrhagic disease detected in Canada were diagnosed in mule deer from Alberta through submissions to the CWHC surveillance program from collaborators. Adenovirus hemorrhagic disease is a highly fatal disease of white-tailed and mule deer. It has been identified as an important cause of deer mortality epidemics in Western Canada and other parts of North America, and can impact other cervid species [52,53]. Additionally, we identified several zoonotic diseases (e.g., brucellosis, anthrax, and bovine tuberculosis) that are of concern to the health of people, of ungulate SAR (e.g., bison), and of the agricultural industry in Canada (S4 Table). Detections of septicemia secondary to *Erysipelothrix rhusiopathiae* infection in muskoxen emphasize the importance of passive disease surveillance as a tool for understanding epizootic events impacting ungulates, which may represent population health threats to many sympatric species, including SAR ([22,54]; S3 Table).

We also identified many non-infectious causes of morbidity and mortality in ungulate species, several of which are due to anthropogenic stressors. Trauma was the COD in 15% of cases. While vehicular trauma occurred most frequently, capture-related trauma (e.g., morbidity and mortality secondary to field anesthesia or the deployment of tracking collars) was diagnosed in a total of 30 individuals. These capture-related mortalities highlight the importance of post-mortem examination to help us inform and improve the techniques we use to capture and monitor ungulate populations in the wild. Additionally, we detected several cases of death due to ruminal acidosis in ungulates given intentional or unintentional access to grain, as well as toxicity secondary to pest-control poisons likely targeting other species (e.g., insecticides, rodenticides). Anthropogenic causes of mortality such as toxin ingestion and road mortality reflect the negative consequences that can occur as habitat fragmentation and urbanization continue to push wildlife and humans together [55]. These cases also highlight broader One Health concerns, as train and car collisions with large ungulates risk human health and can lead to negative perceptions of wildlife [56].

Interestingly, we reported 34 cases of neoplasia across various body systems and origins (e.g., primary and metastatic lesions, including incidences of lymphosarcoma, neuroendocrine carcinoma, plasma cell sarcoma, osteosarcoma, and squamous cell carcinoma), highlighting an under-reported but potentially important cause of mortality in ungulate species [57,58]. We also detected focal temporal trends in non-infectious CODs in caribou, with high proportions of emaciation (2016) and fetal distress (2010–2011; e.g., dystocia, abandonment, stillbirth; Fig 5A). These trends may reflect temporal biases in sample submission to the CWHC, but they may also indicate important but uninvestigated health stressors impacting these submitted caribou, such as temporal climatic variability, decreasing habitat quality, the impact of heavy metal exposure, or undetected infectious causes of reproductive loss [20,22,59].

### Limitations and future directions for improving ungulate disease surveillance in Canada

Effective wildlife disease monitoring requires an integration of passive and active disease surveillance approaches, in addition to general population monitoring [32]. Well-designed passive wildlife disease surveillance programs are integral for long-term population monitoring across taxa and regions [33]. By its very nature, however, passive surveillance is biased by opportunistic sample collection and a lack of denominator data, making epidemiological analyses more challenging [32,33,60]. Active surveillance data allows us to more accurately assess prevalence and incidence in populations of interest, though typically with additional logistical and financial burden [61]. The results of our retrospective passive disease surveillance analysis demonstrate many of these benefits and challenges, and also highlight opportunities for improved collaboration and integration of wildlife disease surveillance programs.

The geographic distribution of ungulate cases submitted to the CWHC illustrates a disjointed surveillance presence across Canada (Fig 1). There are several factors contributing to inconsistent coverage, including human population density, location of regional diagnostic centres, resources to collect and transport carcasses/samples to laboratories, funding for wildlife diagnostic services, and siloed data collection across multiple agencies. We have been successful at capturing wildlife disease events in areas where there are a lot of people present to witness and report those events and when it is relatively easy to collect carcasses for necropsy investigation. However, we clearly lack geographic coverage and sample submission in more remote regions (Fig 1). Furthermore, while the CWHC constitutes both a national collective of wildlife diagnostic laboratories and a national database for collating information, varying levels of participation by provinces and territories due to limited funding, logistical constraints, and/or regionally-focused programs hinders collaboration and data sharing, leading to a less holistic understanding of wildlife health at the national level.

There are also several threats to SAR population and sympatric species that were not captured by our analysis, including the impacts of heavy metal contamination and trace mineral imbalances on caribou health and reproduction [59,62], and the potential impacts of introduced muskoxen on caribou disease burden in northeastern Canada [63–65]. These threats represent broader One Health concerns, as they signify potential health and cultural impacts for Indigenous communities that rely on wild ungulates as an important subsistence resource [5,62]. We also had very limited data on bison captured by our passive disease surveillance program, and few cases of moose submitted from Atlantic Canada, where declining populations are of particular concern [14]. Additionally, only two suspected cases of the introduced parasite *Elaphostrongylus rangiferi* were detected in our twenty year passive surveillance program. Limited sample submission from Newfoundland is a likely factor in the underrepresentation of this pathogen which causes epizootic events of neurological disease in caribou and other ungulate species in Newfoundland [66].

To enhance our understanding of ungulate health in Canada, active surveillance programs must build upon, and be integrated with, passive disease surveillance data. For example, the geographic extent of CWD across the four provinces in Canada where it is now endemic was not captured by our dataset (Fig 2A), as only one of these provinces records passive surveillance findings into the CWHC database. Inclusion of results from the active, hunter-based CWD surveillance programs would have also helped to provide a more detailed and nuanced understanding of the severity of CWD across Canada [27,35]. Similarly, active surveillance programs have been integral in our understanding of the prevalence and incidence of many of the aforementioned ungulate diseases on the Canadian landscape (e.g., *Besnoitia tarandi* [67], winter tick disease [26], brucellosis and bovine tuberculosis [25,68], *Erysipelothrix* sp*.* [69]*,* and trace element deficiencies [70]), as well as species of interest (e.g., understanding the health of reintroduced wood bison [71]). Community-based wildlife health surveillance programs, which incorporate Indigenous and local knowledges and target specific species and/or pathogens on an ongoing basis, are also important contributors that can help fill knowledge gaps, especially across remote regions [72,73]. Finally, our dataset could have been enhanced by the inclusion of data collected through other ongoing passive surveillance programs in Canada that partner with hunters and trappers to monitor wildlife health (e.g., [74]). Passive disease surveillance programs such as the one implemented by the CWHC provide a critical foundation for understanding wildlife health and disease, yet these programs are stronger when they are incorporated into a multi-faceted and integrated surveillance approach across agencies [32]. Canada’s Interagency Surveillance Program for Avian Influenza in Wild Birds provides an excellent example of how the integration of active and passive surveillance data across agencies can lead to the improved understanding of wildlife disease that is necessary to inform disease outbreak responses [75,76].

Our results highlight several infectious and non-infectious health threats facing Canadian ungulate species, yet they also emphasize the limitations of a siloed passive disease surveillance program. Working within these potential limitations, we identified an overall increasing temporal trend in CWD cases across susceptible species in Saskatchewan, as well as an increasing trend in *P. tenuis* in moose cases submitted across endemic regions in Canada over time. Additionally, we detected emerging diseases on the landscape as well as rarely reported non-infectious causes of ungulate morbidity, including neoplasia and toxin exposure. Finally, we provided insight into the spatial distribution of health threats across SAR and sympatric species. To build upon this work and achieve a more holistic and actionable understanding of ungulate health in Canada, a combination of improved funding for wildlife disease surveillance as well as enhanced collaboration and data-sharing across institutions is necessary.

## Supporting information

S1 Table Ungulate cases by sex. All ungulate cases submitted for passive disease surveillance to the Canadian Wildlife Health Cooperative between 2003 and 2022, organized by sex category.
(DOCX)

S2 Table Ungulate cases by age status. All ungulate cases submitted for passive disease surveillance to the Canadian Wildlife Health Cooperative between 2003 and 2022, organized by age category.
(DOCX)

S3 Table Pathogens diagnosed as the causative agent in a primary category of diagnosis of infectious/ inflammatory/ transmissible. Pathogens (n = 57) identified as the causative agent for ungulate cases submitted to the Canadian Wildlife Health Cooperative’s passive disease surveillance program between 2003 and 2022 and assigned a primary category of diagnosis of Infectious/ Inflammatory/ Transmissible. This dataset excludes cases assigned a primary category of diagnosis of Infectious/ Inflammatory/ Transmissible where the primary pathogen was not identified (n = 267) or not applicable (n = 21). Additionally, 33 cases are included where the primary category of diagnosis was emaciation attributed to the parasite *Dermacentor albipictus*, *aka* winter tick.
(DOCX)

S4 Table Federally Reportable or Immediately Notifiable diseases detected. Pathogens detected in ungulate cases submitted for passive disease surveillance to the Canadian Wildlife Health Cooperative between 2003 and 2022 which are federally Reportable or Immediately Notifiable diseases in Canada.
(DOCX)

S5 Table Additional or incidental pathogens detected but not associated with a primary category of diagnosis. Additional pathogens detected in ungulate cases submitted for passive disease surveillance to the Canadian Wildlife Health Cooperative between 2003 and 2022. These pathogens were not associated with the assignation of the primary category of diagnosis for each case, but represent either additional disease burden or incidental findings.
(DOCX)

S1 Methods Additional methods for assigning category of diagnosis, subcategory, and body system, where applicable. (DOCX)

S1 Appendix All raw data used for analysis. (XLSX)
