# Supplementary material for: Twenty years of ungulate disease surveillance by the Canadian Wildlife Health Cooperative (2003–2022)
Source: PLoS One. 2026 Mar 5;21(3):e0343520. doi: 10.1371/journal.pone.0343520 (PMC12962481; doi:10.1371/journal.pone.0343520)
Supplement: S1 Table — All ungulate cases submitted for passive disease surveillance to the Canadian Wildlife Health Cooperative between 2003 and 2022, organized by sex category. (DOCX) [file pone.0343520.s001.docx]

| **S1 Table.** **Ungulate cases by sex.** | | | | |
| --- | --- | --- | --- | --- |
| **Species** | **Female** | **Male** | **Unknown** | **Total** |
| **White-tailed Deer** (*Odocoileus virginianus*) | 309 | 409 | 54 | **772** |
| **Moose** (*Alces americanus*) | 285 | 266 | 101 | **652** |
| **Mule Deer** (*Odocoileus hemionus*) | 172 | 313 | 17 | **502** |
| **Elk** (*Cervus canadensis*) | 96 | 75 | 52 | **223** |
| **Caribou** (*Rangifer tarandus*) | 45 | 37 | 91 | **173** |
| **Bighorn Sheep** (*Ovis canadensis*) | 30 | 20 | 7 | **57** |
| **Pronghorn** (*Antilocapra americana*) | 29 | 21 | 3 | **53** |
| **Bison** (*Bison bison*) | 12 | 27 | 9 | **48** |
| **Muskox** (*Ovibos moschatus*) | 7 | 5 | 19 | **31** |
| **Dall’s sheep** (*Ovis dalli*) | 1 | 6 | 1 | **8** |
| **Mountain Goat** (*Oreamnos americanus*) | 2 | 0 | 2 | **4** |
| **Fallow Deer** (*Dama dama*) | 2 | 0 | 0 | **2** |
| ***Total*** | ***990*** | ***1179*** | ***356*** | ***2525*** |
| ***Proportion (95% CIs)*** | ***39.21***  ***(37.30, 41.14)*** | ***46.69***  ***(44.73,48.66)*** | ***14.10***  ***(12.76,15.52)*** |  |
| All ungulate cases submitted for passive disease surveillance to the Canadian Wildlife Health Cooperative between 2003 and 2022, organized by sex category | | | | |
