# Supplementary material for: Twenty years of ungulate disease surveillance by the Canadian Wildlife Health Cooperative (2003–2022)
Source: PLoS One. 2026 Mar 5;21(3):e0343520. doi: 10.1371/journal.pone.0343520 (PMC12962481; doi:10.1371/journal.pone.0343520)
Supplement: S2 Table — All ungulate cases submitted for passive disease surveillance to the Canadian Wildlife Health Cooperative between 2003 and 2022, organized by age category. (DOCX) [file pone.0343520.s002.docx]

| **S2 Table. Ungulate cases by age status.** | | | | |
| --- | --- | --- | --- | --- |
| **Species** | **Adult** | **Juvenile** | **Unknown** | **Total** |
| **White-tailed Deer** (*Odocoileus virginianus*) | 532 | 162 | 78 | **772** |
| **Moose** (*Alces americanus*) | 378 | 170 | 104 | **652** |
| **Mule Deer** (*Odocoileus hemionus*) | 388 | 89 | 25 | **502** |
| **Elk** (*Cervus canadensis*) | 153 | 19 | 51 | **223** |
| **Caribou** (*Rangifer tarandus*) | 36 | 14 | 123 | **173** |
| **Bighorn Sheep** (*Ovis canadensis*) | 30 | 18 | 9 | **57** |
| **Pronghorn** (*Antilocapra americana*) | 44 | 5 | 4 | **53** |
| **Bison** (*Bison bison*) | 30 | 8 | 10 | **48** |
| **Muskox** (*Ovibos moschatus*) | 10 | 6 | 15 | **31** |
| **Dall’s sheep** (*Ovis dalli*) | 1 | 3 | 4 | **8** |
| **Mountain Goat** (*Oreamnos americanus*) | 2 | 0 | 2 | **4** |
| **Fallow Deer** (*Dama dama*) | 1 | 1 | 0 | **2** |
| ***Total*** | **1605** | **495** | **425** | **2525** |
| ***Proportion (95% CIs)*** | **63.56 (61.65, 65.44)** | **19.60 (18.07, 21.21)** | **16.83 (15.39, 18.35)** |  |
| All ungulate cases submitted for passive disease surveillance to the Canadian Wildlife Health Cooperative between 2003 and 2022, organized by age category | | | | |
