# Supplementary material for: Twenty years of ungulate disease surveillance by the Canadian Wildlife Health Cooperative (2003–2022)
Source: PLoS One. 2026 Mar 5;21(3):e0343520. doi: 10.1371/journal.pone.0343520 (PMC12962481; doi:10.1371/journal.pone.0343520)
Supplement: S3 Table — Pathogens (n = 57) identified as the causative agent for ungulate cases submitted to the Canadian Wildlife Health Cooperative’s passive disease surveillance program between 2003 and 2022 and assigned a primary category of diagnosis of Infectious/ Inflammatory/ Transmissible. This dataset excludes cases assigned a primary category of diagnosis of Infectious/ Inflammatory/ Transmissible where the primary pathogen was not identified (n = 267) or not applicable (n = 21). Additionally, 33 cases are included where the primary category of diagnosis was emaciation attributed to the parasite Dermacentor albipictus, aka winter tick. (DOCX) [file pone.0343520.s003.docx]

| **S3 Table. Pathogens diagnosed as the causative agent in a primary category of diagnosis of infectious/ inflammatory/ transmissible**. | |
| --- | --- |
| **Chronic wasting disease** | **297** |
| Mule Deer | 186 |
| White-Tailed Deer | 91 |
| Elk | 15 |
| Moose | 5 |
| ***Parelaphostrongylus tenuis*** | **277** |
| Moose | 212 |
| Mule Deer | 43 |
| Elk | 19 |
| White-Tailed Deer | 3 |
| ***Fascioloides magna*** | **50** |
| Elk | 37 |
| Moose | 10 |
| White-Tailed Deer | 3 |
| **Brain abscess caused by mixed bacterial infection** | **49** |
| White-Tailed Deer | 32 |
| Mule Deer | 16 |
| Pronghorn | 1 |
| ***Trueperella pyogenes*** | **38** |
| White-Tailed Deer | 14 |
| Pronghorn | 8 |
| Mule Deer | 8 |
| Moose | 3 |
| Caribou | 1 |
| Bighorn Sheep | 1 |
| Dall Sheep | 1 |
| Bison | 1 |
| Muskox | 1 |
| ***Cysticercus* sp*.*** | **35** |
| Moose | 29 |
| Caribou | 4 |
| Mule Deer | 1 |
| White-Tailed Deer | 1 |
| **Emaciation secondary to winter tick burden (*Dermacentor albipictus*)*** | **33** |
| Moose | 33 |
| **Mixed bacterial infection** | **28** |
| White-Tailed Deer | 8 |
| Moose | 6 |
| Pronghorn | 5 |
| Caribou | 3 |
| Mule Deer | 3 |
| Muskox | 1 |
| Elk | 1 |
| Bighorn Sheep | 1 |
| ***Echinococcus* sp*.*** | **25** |
| Moose | 20 |
| Caribou | 2 |
| Elk | 2 |
| White-Tailed Deer | 1 |
| **Cutaneous fibropapilloma (presumed or confirmed papillomavirus cause)** | **21** |
| White-Tailed Deer | 9 |
| Moose | 8 |
| Mule Deer | 4 |
| ***Besnoitia tarandi* (confirmed or presumed)** | **16** |
| Caribou | 16 |
| ***Brucella suis* (confirmed and suspected)**** | **15** |
| Caribou | 9 |
| Muskox | 3 |
| Bison | 3 |
| ***Clostridium* sp.** | **15** |
| White-Tailed Deer | 9 |
| Moose | 2 |
| Elk | 2 |
| Bison | 1 |
| Pronghorn | 1 |
| ***Sarcocyst* sp.** | **14** |
| Moose | 5 |
| White-Tailed Deer | 3 |
| Caribou | 3 |
| Elk | 2 |
| Bison | 1 |
| **Foot rot caused by mixed bacterial infection** | **14** |
| Caribou | 9 |
| White-Tailed Deer | 3 |
| Bighorn Sheep | 1 |
| Elk | 1 |
| ***Fusobacterium* sp.** | **12** |
| White-Tailed Deer | 4 |
| Mule Deer | 4 |
| Pronghorn | 3 |
| Moose | 1 |
| **Anthrax (*Bacillus anthracis*; confirmed cases)** | **11** |
| Bison | 9 |
| White-Tailed Deer | 1 |
| Moose | 1 |
| ***Streptococcus* sp.** | **10** |
| Moose | 3 |
| Caribou | 2 |
| White-Tailed Deer | 2 |
| Pronghorn | 2 |
| Bighorn Sheep | 1 |
| **Epizootic hemorrhagic disease virus**** | **9** |
| White-Tailed Deer | 9 |
| ***Parelaphostrongylus odocoilei*** | **9** |
| Mule Deer | 9 |
| ***Escherichia coli*** | **8** |
| Caribou | 3 |
| Moose | 4 |
| Mule Deer | 1 |
| ***Dermatomyces* sp. (ringworm)** | **7** |
| Mule Deer | 6 |
| White-Tailed Deer | 1 |
| ***Pasteurella multocida*** | **7** |
| Moose | 3 |
| White-Tailed Deer | 1 |
| Mule Deer | 1 |
| Elk | 1 |
| Bison | 1 |
| **Orf/contageous ecthyema (presumed or confirmed parapoxvirus cause)** | **7** |
| Muskox | 4 |
| Mountain Goat | 2 |
| Bighorn Sheep | 1 |
| ***Erysipelothrix rhusiopathiae*** | **6** |
| Muskox | 6 |
| ***Demodex* sp.** | **5** |
| White-Tailed Deer | 4 |
| Mule Deer | 1 |
| **Lungworm - unknown species** | **5** |
| Mule Deer | 3 |
| Bighorn Sheep | 1 |
| Dall Sheep | 1 |
| ***Staphylococcus* sp.** | **5** |
| Elk | 2 |
| Moose | 1 |
| Dall Sheep | 1 |
| White-Tailed Deer | 1 |
| **Bovine tuberculosis (*Mycobacterium bovis*)** | **4** |
| Elk | 4 |
| ***Setaria* sp.** | **4** |
| Moose | 3 |
| Caribou | 1 |
| ***Dermatophilus congolensis*** | **4** |
| White-Tailed Deer | 4 |
| **Malignant Catarrhal Fever**** | **4** |
| Moose | 3 |
| White-Tailed Deer | 1 |
| ***Dictyocaulus viviparous*** | **3** |
| Elk | 2 |
| Moose | 1 |
| ***Corynebacterium* sp.** | **3** |
| Moose | 1 |
| White-Tailed Deer | 1 |
| Pronghorn | 1 |
| ***Moraxella* sp.** | **3** |
| Moose | 2 |
| Mule Deer | 1 |
| ***Psoroptes* sp.** | **3** |
| Bighorn Sheep | 3 |
| ***Elaphostrongylus rangiferi -* suspected** | **2** |
| Moose | 1 |
| Caribou | 1 |
| ***Listeria monocytogenes*** | **2** |
| White-Tailed Deer | 2 |
| ***Aspergillus* sp.** | **2** |
| White-Tailed Deer | 1 |
| Mule Deer | 1 |
| ***Bibersteinia trehalosi*** | **2** |
| White-Tailed Deer | 1 |
| Bighorn Sheep | 1 |
| **Adenovirus** | **2** |
| Mule Deer | 2 |
| ***Pseudomonas* sp.** | **2** |
| Moose | 2 |
| ***Hypoderma tarandi*** | **1** |
| Caribou | 1 |
| ***Taenia arctos*** | **1** |
| Moose | 1 |
| ***Fasciola* sp.** | **1** |
| Elk | 1 |
| ***Mannheimia* sp.** | **1** |
| White-Tailed Deer | 1 |
| ***Cryptococcus gattii VGIIb*** | **1** |
| White-Tailed Deer | 1 |
| ***Yersinia pseudotuberculosis*** | **1** |
| Bighorn Sheep | 1 |
| ***Klebsiella oxytoca*** | **1** |
| Caribou | 1 |
| **Mycobacterium aviam sp. Paratuberculosis (Johne's Disease)** | **1** |
| White-Tailed Deer | 1 |
| ***Proteus mirabilis*** | **1** |
| Moose | 1 |
| ***Mycoplasma bovis*** | **1** |
| White-Tailed Deer | 1 |
| ***Protostrongylus stilesi*** | **1** |
| Dall Sheep | 1 |
| ***Babesia odocoilei*** | **1** |
| White-Tailed Deer | 1 |
| ***Actinomyces* sp.** | **1** |
| Bison | 1 |
| ***Onchocerca* sp.** | **1** |
| Caribou | 1 |
| **Herpesvirus sp.** | **1** |
| Mule Deer | 1 |
| Pathogens (n=57) identified as the causative agent for ungulate cases submitted to the Canadian Wildlife Health Cooperative’s passive disease surveillance program between 2003 and 2022 and assigned a primary category of diagnosis of Infectious/ Inflammatory/ Transmissible. This dataset excludes cases assigned a primary category of diagnosis of Infectious/ Inflammatory/ Transmissible where the primary pathogen was not identified (n=267) or not applicable (n=21). Additionally, 33 cases are included where the primary category of diagnosis was emaciation attributed to the parasite *Dermacentor albipictus*, aka winter tick  *Primary category of diagnosis = emaciation | |
| **Various types/strains/serovars | |
