# Supplementary material for: Twenty years of ungulate disease surveillance by the Canadian Wildlife Health Cooperative (2003–2022)
Source: PLoS One. 2026 Mar 5;21(3):e0343520. doi: 10.1371/journal.pone.0343520 (PMC12962481; doi:10.1371/journal.pone.0343520)
Supplement: S4 Table — Pathogens detected in ungulate cases submitted for passive disease surveillance to the Canadian Wildlife Health Cooperative between 2003 and 2022 which are federally Reportable or Immediately Notifiable diseases in Canada. (DOCX) [file pone.0343520.s004.docx]

| **S4 Table. Federally Reportable or Immediately Notifiable diseases detected.** | | | |
| --- | --- | --- | --- |
| **Pathogens of Interest** | **Number of Cases** | **Species** | **Additional suspected cases** |
| Chronic Wasting Disease^1^ | 297 | Mule Deer (186), White-tailed Deer (91), Elk (15), Moose (5) |  |
| *Besnoitia* *tarandi* (confirmed or presumed)^2^ | 16 | Caribou (16) | Caribou=4, Dall’s sheep=1 |
| Anthrax (*Bacillus anthracis*)^1^ | 11 | Bison (9), White-tailed Deer (1), Moose (1) | Bison=53, Moose=1 |
| *Brucella* suis*^1^ (biovar 4 confirmed or presumed) | 11 | Caribou (9), Muskox (2) | Caribou=2, Muskox=1, Bison=1 (*Brucella* species not identified) |
| Epizootic Hemorrhagic Disease Virus*^2^ | 9 | White-tailed Deer (9) | *Alberta 2013 cluster* – 50+ in White-tailed Deer + 3 in Pronghorn cases.  *Ontario 2021 cluster* – 35+ in White-tailed Deer. |
| Bovine tuberculosis (*Mycobacterium bovis*)^1^ | 4 | Elk (4) |  |
| Pathogens detected in ungulate cases submitted for passive disease surveillance to the Canadian Wildlife Health Cooperative between 2003 and 2022 which are federally Reportable or Immediately Notifiable diseases in Canada  1 = Federally Reportable disease in Canada | | | |
| 2= Federally Immediately Notifiable disease in Canada | | |  |
| *Various types/strains/serovars | | |  |
