# Supplementary material for: Twenty years of ungulate disease surveillance by the Canadian Wildlife Health Cooperative (2003–2022)
Source: PLoS One. 2026 Mar 5;21(3):e0343520. doi: 10.1371/journal.pone.0343520 (PMC12962481; doi:10.1371/journal.pone.0343520)
Supplement: S5 Table — Additional pathogens detected in ungulate cases submitted for passive disease surveillance to the Canadian Wildlife Health Cooperative between 2003 and 2022. These pathogens were not associated with the assignation of the primary category of diagnosis for each case, but represent either additional disease burden or incidental findings. (DOCX) [file pone.0343520.s005.docx]

| **S5 Table. Additional or incidental pathogens detected but not associated with a primary category of diagnosis.** | |
| --- | --- |
| ***Additional Pathogens Detected*** | **Number of cases** |
| *Sarcocyst* sp. | 115 |
| Lungworm - unknown sp. | 39 |
| Winter tick - unknown significance | 35 |
| *Trueperella pyogenes* | 32 |
| *Parelaphostrongylus tenuis –* unknown significance/incidental | 27 |
| *Cysticercus* sp. | 24 |
| *Dictyocaulus* sp. | 13 |
| *Fascioloides magna* | 12 |
| *Nematodes - unknown* sp. | 12 |
| Cutaneous fibropapilloma (presumed or confirmed papillomavirus cause) | 19 |
| *Cestodes -* unknown sp. | 10 |
| Nasal bots - various sp. | 10 |
| *Echinococcus* sp. | 8 |
| *Fusobacterium* sp. | 8 |
| *Setaria* sp. | 8 |
| *Streptococcus* sp. | 7 |
| *Umingmakstrongylus pallikuukensis* | 7 |
| *Clostridium* sp. | 6 |
| *Escherichia coli* | 6 |
| *Besnoitia* sp. | 5 |
| *Dermatomycosis* sp. (ringworm) | 4 |
| *Staphylococcus* sp. | 4 |
| Lice sp. | 3 |
| Liver fluke - unknown sp. | 3 |
| *Pasteurella* sp. | 3 |
| *Protostrongylus* sp. *- suspected* | 3 |
| *Elaeophora schneideri* | 2 |
| *Escherichia* sp. | 2 |
| *Onchocerca cervipedis* | 2 |
| *Parelaphostrongylus odocoilei* | 2 |
| *Actinomyces* sp. | 1 |
| *Bacteroides fragilis* | 1 |
| *Bibersteinia trehalosi* | 1 |
| *Candida* sp. | 1 |
| *Capillaria* sp. | 1 |
| *Dicrocoelium dendriticum* | 1 |
| *Enterococcus* sp. | 1 |
| *Listeria monocytogenes* | 1 |
| *Marshallagia marshalli* | 1 |
| *Mycoplasma ovipneumoniae* | 1 |
| *Neorickettsia helminthoeca* | 1 |
| Orf/contageous ecthyema (presumed or confirmed parapoxvirus cause) | 1 |
| *Pantoea agglomerans* | 1 |
| *Parelaphostrongylus andersoni - incidental* | 1 |
| *Prevotella melaninogenica* | 1 |
| *Taenia solium* | 1 |
| *Tricholipeurus* sp. | 1 |
| *Trichuris* sp. | 1 |
| *Wohlfahrtilmnas chitiniclastica* | 1 |
| *Wyominia tetoni* | 1 |
| Additional pathogens detected in ungulate cases submitted for passive disease surveillance to the Canadian Wildlife Health Cooperative between 2003 and 2022. These pathogens were not associated with the assignation of the primary category of diagnosis for each case, but represent either additional disease burden or incidental findings | |
