# Supplementary material for: Twenty years of ungulate disease surveillance by the Canadian Wildlife Health Cooperative (2003–2022)
Source: PLoS One. 2026 Mar 5;21(3):e0343520. doi: 10.1371/journal.pone.0343520 (PMC12962481; doi:10.1371/journal.pone.0343520)
Supplement: S1 Methods — (DOCX) [file pone.0343520.s006.docx]

**S1 Methods. Additional methods for assigning category of diagnosis, subcategory, and body system, where applicable.**

***Category of diagnosis:***

The category of diagnosis “Normal”, included cases where the pathologist had sufficient samples and history to determine that the animal appeared healthy at the time of death. For example, these were often hunter-killed animals where the hunter had concerns at field-dressing and requested additional testing (i.e. found a liver lesion which the pathologist determined was a normal finding), or cases where the investigating conservation officer needed necropsy confirm the cause of death as gunshot and the animal was indeed deemed healthy at time of gunshot by the pathologist. Importantly, normal cases never included an abnormal history (i.e. a neurological animal would never be classified as “Normal”, even if the samples submitted were normal), and any ancillary testing done in these cases also had to be within normal limits before the classification was applied.

“Normal” cases do not include hunter-killed animals that were automatically screened for chronic wasting disease (CWD) but found to be negative, as this was part of the active surveillance program and these were screened out of our data.

***Body system categories:***

o Multi-system

o Digestive System - including pancreas

o Endocrine System

o Hepatobiliary System

o Integumentary System

o Urinary System

o Cardiovascular System

o Eye/Ear

o Lymphoid system – including bone marrow, spleen, lymph node, tonsils, thymus

o Respiratory system

o Musculoskeletal System

o Nervous System – including central and peripheral

o Reproductive System

**Additional subcategory definitions (where applicable):**

- ***Primary COD – Other:***
  - Subcategory: Fetal distress
    - Abandonment, dystocia, stillbirth
  - Subcategory: Neoplasia
    - Various, based on pathologist interpretation of ancillary testing
  - Subcategory: Nutritional
    - Abomasal impaction, frothy bloat, ruminal acidosis (i.e. grain overload), left displaced abomasum, polioencephalomalacia
  - Subcategory: Other
    - Various
  - Subcategory: Toxicity
    - Insecticide (carbamate or organophosphate), nitrate, rodenticide, salt toxicity, Se toxicity, Cu toxicity, strychnine
